# LINC00973/DTX3L Axis Promotes Non‐Small Cell Lung Cancer Progression and Serves as a Therapeutic Target

**DOI:** 10.1002/smmd.70029

**Published:** 2026-02-09

**Authors:** Yanke Chen, Yu Qian, Jiayuan Shi, Yi Wang, Tingyu Fu, Shuting Meng, Maoye Wang, Min Fu, Jiahui Zhang, Xiaoxin Zhang, Runbi Ji, Jianmei Gu, Xu Zhang, Zhe‐Sheng Chen, Xiuqin Ma, Xinjian Fang

**Affiliations:** ^1^ Department of Laboratory Medicine, School of Medicine Jiangsu University Zhenjiang China; ^2^ Department of Medical Laboratory Wuxi Traditional Chinese Medicine Hospital Wuxi China; ^3^ Department of Clinical Laboratory Medicine Nantong Tumor Hospital/Affiliated Tumor Hospital Nantong University Nantong China; ^4^ College of Pharmacy and Health Sciences St. John’s University Queens New York USA; ^5^ Department of Pulmonary and Critical Care Medicine Affiliated Yixing Hospital of Jiangsu University Yixing China; ^6^ Department of Oncology Affiliated Gaochun Hospital of Jiangsu University Nanjing China

**Keywords:** DTX3L, LINC00973, non‐small cell lung cancer, targeted therapy

## Abstract

Long non‐coding RNAs (lncRNAs) constitute a critical class of regulatory molecules involved in cancer biology and play pivotal roles in tumor initiation and progression. Nevertheless, the biological functions of many newly identified lncRNAs in non‐small cell lung cancer (NSCLC), as well as their potential therapeutic relevance, remain insufficiently characterized. In this study, high‐throughput sequencing analysis of paired NSCLC tumor tissues and adjacent non‐tumorous samples revealed that LINC00973 is significantly upregulated in tumor specimens. Moreover, elevated LINC00973 expression was found to be closely associated with poor clinical outcomes in patients with NSCLC. Functional assays showed that LINC00973 knockdown inhibits NSCLC cell proliferation, migration, and invasion while inducing apoptosis, whereas overexpression produces opposite effects. These observations were confirmed in vivo, where LINC00973 depletion markedly suppressed tumor growth and metastasis. Mechanistically, LINC00973 interacts with and stabilizes deltex E3 ubiquitin ligase 3L (DTX3L), preventing its ubiquitination‐mediated degradation and activating the AKT signaling pathway. Therapeutically, RGD‐modified exosome‐mediated delivery of LINC00973 siRNA significantly inhibited NSCLC progression in mouse models. Moreover, a synthetic biology‐based strategy enabling hepatic production of exosomes carrying LINC00973‐targeting siRNA achieved robust anti‐tumor effects. Together, these findings establish LINC00973 as an oncogenic lncRNA that promotes NSCLC progression via DTX3L stabilization and highlight LINC00973 as a promising therapeutic target.

## Key Points

- LINC00973 plays an oncogenic role in NSCLC progression and its upregulation predicts poor prognosis.
- LINC00973 binds to and stabilizes DTX3L protein, activating the AKT signaling pathway.
- Targeted inhibition of LINC00973 via in vitro fabricated and in vivo self‐assembled exosome delivery of siRNA represents a promising therapeutic strategy in NSCLC.

## Graphical abstract

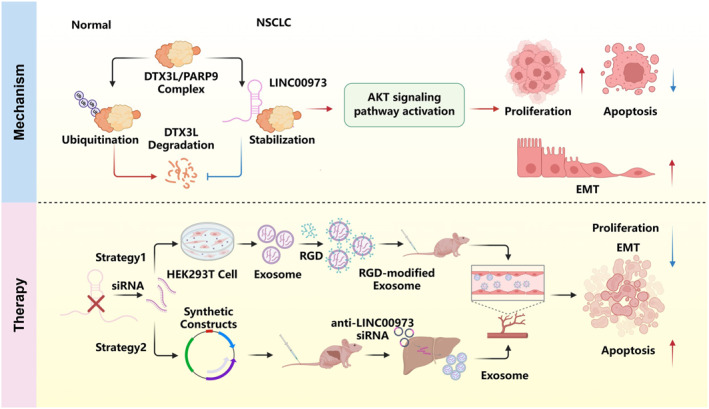

## 1 Introduction

Lung cancer remains the leading cause of cancer‐related incidence and mortality worldwide, with non‐small cell lung cancer (NSCLC) accounting for approximately 85% of all lung cancer cases [1]. Recent advances in diagnostic and surgical procedures, as well as medical therapies targeted against the most common driver mutations, have improved the survival rate of NSCLC. Nevertheless, the mortality rate remains high, and effective molecular therapeutic targets are still limited in routine clinical practice [1]. Therefore, in order to identify novel therapeutic targets and ultimately improve treatment outcomes, there is an urgent need to elucidate the underlying molecular mechanisms driving NSCLC progression.

Accumulating evidence demonstrates that long non‐coding RNAs (lncRNAs) play pivotal roles in a wide range of physiological processes and pathological conditions in humans [2, 3, 4]. LncRNAs regulate gene expression at both transcriptional and post‐transcriptional levels through interactions with nucleic acids and proteins, thereby orchestrating complex regulatory networks that govern essential cellular processes, including proliferation, apoptosis, and differentiation [5, 6]. In addition, lncRNAs can be packaged into exosomes and transferred between cells, enabling their active participation in intercellular communication and contributing to cancer initiation and progression [7, 8]. The multifaceted involvement of lncRNAs in the development and progression of NSCLC has become increasingly evident [9, 10, 11, 12]. For example, lncRNA HIF1A‐AS2 and MYC form a reciprocal positive feedback loop that promotes cell proliferation and metastasis in KRAS‐driven NSCLC [13]. Consequently, elucidating the molecular mechanisms underlying lncRNA function is of considerable importance, as it may facilitate the identification of novel biomarkers and therapeutic targets for clinical application.

In recent years, extracellular vesicles have attracted considerable interest as innovative platforms for drug delivery, largely because of their excellent biocompatibility, inherent cell‐targeting properties, and efficient cargo‐loading capacity [14]. Through specific genetic or chemical modifications, these vesicles can be engineered to enhance their recognition of and binding to target cells while minimizing off‐target toxicity to normal tissues [14, 15, 16, 17]. As a result, exosomes represent a promising delivery system for therapeutic agents in cancer treatment [18, 19, 20]. However, the clinical translation of exosome‐based therapies continues to face substantial challenges, including difficulties in large‐scale production, batch‐to‐batch variability, and the lack of standardized regulatory guidelines [15, 21, 22]. To overcome these limitations, recent studies have proposed the development of engineered exosome systems capable of efficiently transporting therapeutic molecules. Interestingly, Sun et al. reported an innovative strategy using synthetic biology to generate small interfering RNAs (siRNAs) packaged within self‐assembled exosomes in vivo [23]. This engineered construct incorporates a pre‐miR‐155 backbone, a cytomegalovirus (CMV)‐driven promoter, and an anti‐lncRNA siRNA sequence. Following processing in mouse hepatocytes, the construct facilitates the production of exosomes containing the anti‐lncRNA siRNA, which subsequently enter the circulation and are delivered to tumor tissues. Upon uptaken by cancer cells, the released siRNA suppresses tumor growth. Importantly, this approach effectively addresses the key challenges associated with large‐scale exosome production and quality control, thereby providing a novel and promising avenue for cancer therapy.

In this study, we identified a new lncRNA (LINC00973) that is markedly upregulated in NSCLC tissues. Subsequent in vitro and in vivo experiments revealed that LINC00973 promotes NSCLC growth and metastasis. Mechanistically, LINC00973 exerts its oncogenic function through direct interaction with Deltex E3 ubiquitin ligase 3L (DTX3L), thereby disrupting DTX3L ubiquitination and preventing its proteasomal degradation. The resulting stabilization of DTX3L leads to aberrant activation of the AKT pathway, which facilitates NSCLC progression. Moreover, we demonstrated that targeted silencing of LINC00973 using exosome‐mediated delivery of siRNA significantly suppresses malignant phenotypes in NSCLC. In addition, employing a synthetic biology‐based strategy, we successfully generated self‐assembled siRNA‐loaded exosomes in the mouse liver that specifically target LINC00973, exhibiting a strong therapeutic potential to inhibit NSCLC progression.

## 2 Results

### 2.1 LINC00973 Is Upregulated in NSCLC and Related to Poor Prognosis

**FIGURE 1:**
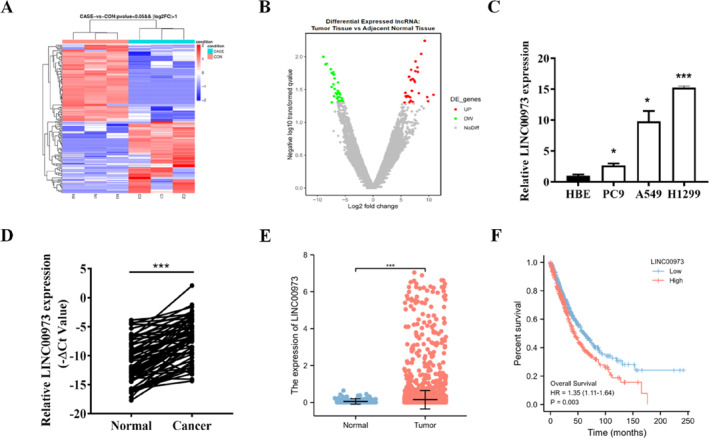
LINC00973 is upregulated in NSCLC and related to poor prognosis. (A) Heatmap showing the expression profiles of differentially expressed lncRNAs based on RNA‐sequencing data from paired NSCLC tumor tissues and adjacent normal tissues. (B) Volcano plot illustrating the distribution of differentially expressed lncRNAs. (C) qRT‐PCR analysis of LINC00973 expression in NSCLC cell lines (A549, H1299, and PC9) and the normal HBE cell line. (D) qRT‐PCR analysis of LINC00973 expression in paired tumor and adjacent normal tissues from 61 NSCLC patients. (E) Differential expression of LINC00973 in tumor and normal tissues of NSCLC patients based on TCGA data. (F) Kaplan–Meier survival analysis showing the association between LINC00973 expression levels and overall survival in NSCLC patients. Statistical significance was assessed using the log‐rank test. Data are presented as mean ± SD. *p < 0.05; ***p < 0.001.

To investigate the potential involvement of lncRNAs in the pathogenesis of NSCLC, we conducted high‐throughput RNA sequencing in paired tumor and adjacent non‐tumorous tissue samples obtained from patients with NSCLC (Figure 1A). Among the 173 differentially expressed lncRNAs (fold change > 2 and *p* value < 0.05), 84 lncRNAs were upregulated, and 89 lncRNAs were downregulated in the NSCLC tissues relative to the matched normal samples (Figure 1B). Notably, LINC00973 exhibited the most pronounced upregulation among all aberrantly expressed lncRNAs identified in the initial screening analysis (Supporting Information S1: Figure S1A) and was therefore selected for further functional characterization.

We next assessed the expression profile of LINC00973 in NSCLC cell lines and clinical specimens. Quantitative analysis revealed that LINC00973 expression was markedly elevated in the NSCLC cell lines A549, H1299, and PC9 compared with human bronchial epithelial (HBE) cells (Figure 1C). Consistently, examination of 61 paired NSCLC tumor and adjacent normal tissue samples demonstrated a significant increase in LINC00973 expression in tumor tissues relative to their corresponding normal counterparts (Figure 1D and Supporting Information S1: Figure S1B). To determine the clinical relevance of LINC00973, we further analyzed its expression across different pathological stages of NSCLC. Interestingly, the expression level of LINC00973 did not progressively increase with advancing tumor stage (Supporting Information S1: Figure S1C).

In addition, analysis of the TCGA dataset confirmed the elevated expression of LINC00973 in NSCLC tumor tissues, yielding results consistent with those obtained from our clinical cohort (Figure 1E and Supporting Information S1: Figure S1D). Kaplan–Meier survival analysis further demonstrated that patients with high LINC00973 expression exhibited significantly shorter overall survival compared with those expressing lower levels of this lncRNA (Figure 1F). Collectively, these findings indicate that LINC00973 is aberrantly overexpressed in NSCLC and that its elevated expression is closely associated with poor clinical prognosis.

### 2.2 LINC00973 Promotes NSCLC Progression

**FIGURE 2:**
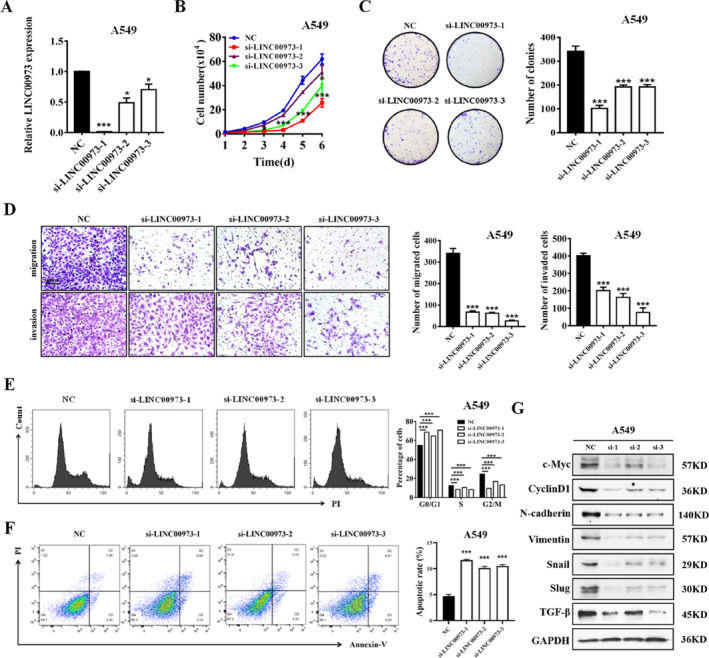
LINC00973 knockdown suppresses NSCLC progression. (A) qRT‐PCR analysis confirming the efficiency of LINC00973 knockdown in NSCLC cells. (B) Cell proliferation was assessed using the CCK‐8 assay following LINC00973 silencing. (C) Colony formation assays showing the clonogenic ability of NSCLC cells after LINC00973 knockdown. (D) Transwell migration and Matrigel invasion assays evaluating the migratory and invasive capacities of NSCLC cells with LINC00973 depletion. (E) Flow cytometric analysis of cell cycle distribution in NSCLC cells after LINC00973 knockdown. (F) Flow cytometric analysis of apoptosis in NSCLC cells following LINC00973 silencing. (G) Western blot analysis of proteins related to cell proliferation and EMT in NSCLC cells after LINC00973 depletion. Data are presented as mean ± SD (*n* = 3, *p < 0.05; **p < 0.01; ***p < 0.001).

Given the marked upregulation of LINC00973 in tumor tissues compared with non‐tumorous counterparts, we next investigated its functional role in NSCLC progression by gain‐ and loss‐of‐function studies. Efficient knockdown of LINC00973 was first confirmed by quantitative real‐time PCR (qRT‐PCR; Figure 2A and Supporting Information S1: Figure S2A). Functional assays revealed that LINC00973 silencing significantly suppressed NSCLC cell proliferation and colony‐forming ability (Figure 2B,C and Supporting Information S1: Figure S2B,C). In addition, depletion of LINC00973 markedly reduced the migratory and invasive capacities of NSCLC cells (Figure 2D and Supporting Information S1: Figure S2D). Flow cytometric analysis further demonstrated that LINC00973 knockdown induced G1 phase cell‐cycle arrest, accompanied by a reduction in the proportion of cells in S phase (Figure 2E and Supporting Information S1: Figure S2E) as well as a pronounced increase in apoptotic cell populations (Figure 2F and Supporting Information S1: Figure S2F). Consistently, suppression of LINC00973 resulted in decreased expression of proliferation‐associated proteins, including c‐Myc and cyclin D1, along with key epithelial‐mesenchymal transition (EMT)‐related markers such as N‐cadherin, vimentin, Snail, Slug, and TGF‐β (Figure 2G and Supporting Information S1: Figure S2G). To further substantiate the oncogenic role of LINC00973, gain‐of‐function experiments were conducted by overexpressing LINC00973 in PC9 cells (Supporting Information S1: Figure S3A–E). Collectively, these findings demonstrate that LINC00973 functions as an oncogenic lncRNA that promotes the malignant progression of NSCLC.

### 2.3 LINC00973 Interacts With DTX3L and Activates AKT Signaling Pathway

**FIGURE 3:**
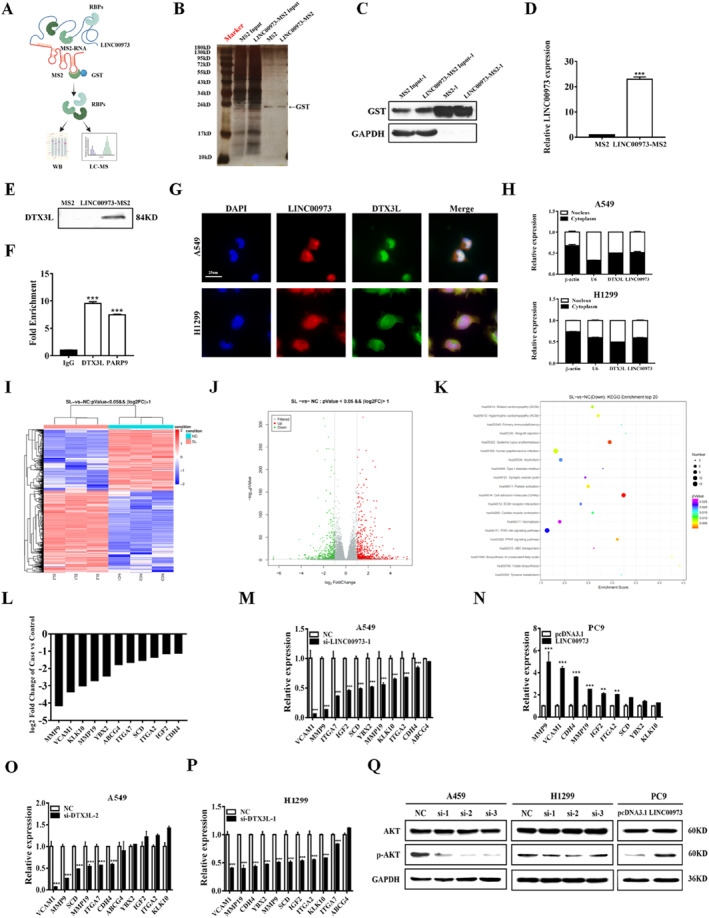
LINC00973 interacts with DTX3L protein and regulates the AKT pathway in NSCLC cells. (A) Schematic representation of the TRAP experiment. (B and C) Silver staining (B) and Western blot analysis (C) confirm GST expression in TRAP‐processed samples. (D) qRT‐PCR analysis of LINC00973 enrichment in TRAP‐pulled‐down products. (E) TRAP followed by Western blot analysis validating the interaction between LINC00973 and the DTX3L protein. (F) RIP assays showing the association of LINC00973 with DTX3L and PARP9 proteins. (G and H) Subcellular localization and co‐localization of LINC00973 and DTX3L in NSCLC cells assessed by RNA‐FISH combined with immunofluorescence (G) and subcellular fractionation analysis (H). (I and J) Heatmap (I) and volcano plot (J) displaying differentially expressed genes between control and LINC00973‐depleted A549 cells. (K) Pathway enrichment analysis of differentially expressed genes following LINC00973 knockdown. (L) Representative downregulated genes identified by RNA‐sequencing in A549 cells after LINC00973 depletion. (M and N) qRT‐PCR validation of selected differentially expressed genes in NSCLC cells following LINC00973 modulation and DTX3L‐related analyses. (O and P) qRT‐PCR analysis of differentially expressed genes in NSCLC cells after DTX3L silencing. (Q) Western blot analysis of AKT pathway‐related proteins in NSCLC cells with LINC00973 knockdown or overexpression (*n* = 3, **p < 0.01; ***p < 0.001).

Previous studies have shown that lncRNAs can interact with proteins to form functional ribonucleoprotein complexes, thereby modulating intracellular signaling pathways and regulating downstream gene expression [24]. To elucidate the molecular mechanism through which LINC00973 contributes to NSCLC progression, a tagged RNA affinity purification (TRAP) strategy was first employed to identify proteins associated with LINC00973. In particular, we co‐transfected the LINC00973‐MS2 vector with the GST‐MS2 vector into A549 cells and obtained the potential binding proteins of LINC00973 by GST pull‐down combined with LC‐MS/MS analysis (Figure 3A–D). Among these candidates, the E3 ubiquitin ligase complex composed of DTX3L and PARP9 exhibited the highest abundance (Supporting Information S1: Table S1). Given that DTX3L serves as an E3 ubiquitin ligase with a critical role in protein turnover and signal transduction, and that the DTX3L‐PARP9 complex emerged as a prominent interacting partner, this complex was selected for further mechanistic investigation. Further validation experiments by TRAP and Western blot analysis confirmed that LINC00973 exhibited strong binding ability to the DTX3L protein (Figure 3E). In addition, RNA immunoprecipitation (RIP) assays demonstrated that LINC00973 was significantly enriched in immunoprecipitate obtained with antibodies against both DTX3L and PARP9 in NSCLC cells (Figure 3F). In addition, both immunofluorescent staining and cell fraction studies revealed co‐localization of LINC00973 and DTX3L within NSCLC cells (Figure 3G,H).

To delineate the signaling pathways regulated by LINC00973, RNA sequencing was performed in NSCLC cells following LINC00973 knockdown and in corresponding control cells, followed by clustering and pathway analysis of gene expression profiles (Figure 3I,J). Comparative analysis revealed that LINC00973 depletion resulted in the upregulation of 677 genes and the downregulation of 508 genes relative to control cells. Consistent with these global changes, several oncogenic factors, including MMP9 and VCAM1, were markedly reduced in LINC00973‐deficient NSCLC cells (Figure 3L). The expression patterns of selected differentially expressed genes were further validated by qRT‐PCR in NSCLC cells subjected to LINC00973 knockdown or overexpression (Figure 3M,N). Notably, a similar gene expression profile was observed in NSCLC cells following DTX3L depletion (Figure 3O,P), supporting a functional link between LINC00973 and DTX3L‐mediated regulatory networks. Pathway enrichment analysis demonstrated that the differentially expressed genes were predominantly enriched in cancer‐related signaling cascades, with particular emphasis on the AKT signaling pathway (Figure 3K). Importantly, transcriptomic profiling identified the AKT pathway as one of the most significantly affected pathways following LINC00973 suppression, highlighting its potential role as a downstream effector. This observation is further supported by previous reports demonstrating a critical role for DTX3L in AKT pathway regulation [25]. Based on these findings, the AKT signaling axis was selected for further validation. Western blot analysis revealed that LINC00973 knockdown significantly reduced the levels of *p*‐AKT in NSCLC cells, whereas ectopic overexpression of LINC00973 produced the opposite effect (Figure 3Q). Collectively, these results indicate that LINC00973 positively regulates AKT signaling in NSCLC cells, thereby contributing to tumor progression.

### 2.4 DTX3L Regulates AKT Pathway and Promotes NSCLC Progression

**FIGURE 4:**
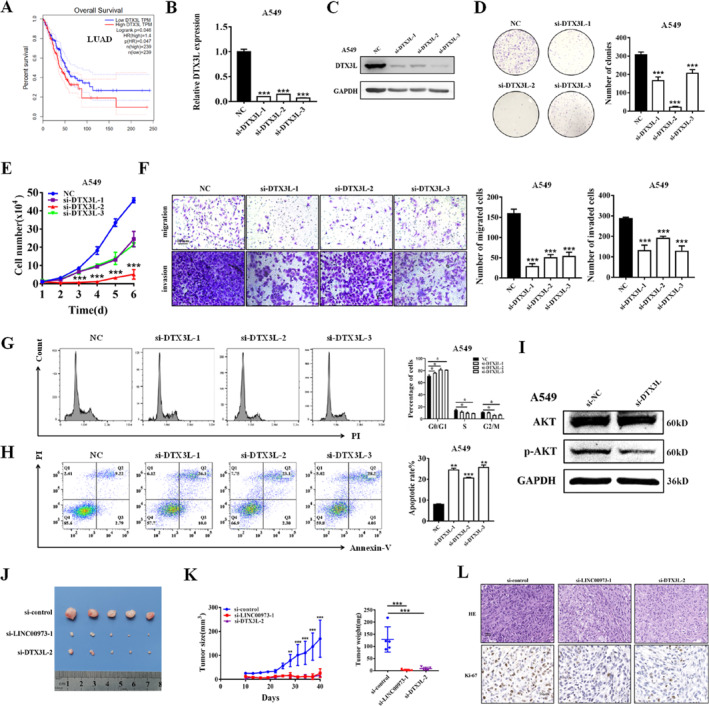
DTX3L regulates the AKT pathway and promotes NSCLC progression. (A) Kaplan–Meier survival analysis showed the correlation between DTX3L expression and overall survival of NSCLC patients based on GEPIA database data. (B and C) qRT‐PCR (B) and Western blot (C) analyses confirmed the efficiency of DTX3L knockdown at the mRNA and protein levels, respectively. (D) Colony formation assays evaluated the clonogenic capacity of NSCLC cells following DTX3L depletion. (E) Cell proliferation of NSCLC cells with DTX3L knockdown assessed by the CCK‐8 assay. (F) Transwell migration and Matrigel invasion assays performed in NSCLC cells after DTX3L silencing. (G) Flow cytometric analysis of apoptosis in NSCLC cells following DTX3L knockdown. (H) Flow cytometric analysis of cell cycle distribution in NSCLC cells after DTX3L depletion. (I) Western blot analysis of AKT signaling pathway‐related proteins in NSCLC cells with DTX3L silencing compared with control cells. (J) Representative images of tumor tissues from each experimental group in the xenograft model. (K) Tumor growth curves and final tumor weights measured for each experimental group. (L) Representative images of hematoxylin and eosin (H&E) and Ki‐67 immunohistochemical staining of tumor tissues. Data are shown as means ± SD (*p < 0.05; **p < 0.01; ***p < 0.001).

To assess the potential clinical significance of DTX3L in NSCLC, its expression was initially analyzed using the TCGA dataset. Elevated DTX3L expression was found to correlate with poor overall survival in NSCLC patients, suggesting its potential as a prognostic marker (Figure 4A and Supporting Information S1: Figure S4A,B). We validated the protein‐level relevance of this axis in clinical specimens using immunohistochemistry (IHC) for DTX3L and *p*‐AKT on serial patient sections. A robust positive correlation was observed between their expression levels. A pronounced increase in the proportion of positive cells for both markers was also evident in advanced‐stage (III/IV) versus stage I tumors (Figure 4C). Based on this clinical correlation, we performed gain‐ and loss‐of‐function experiments to analyze the biological roles of DTX3L in NSCLC progression. Knockdown of DTX3L effectively reduced its mRNA and protein levels in NSCLC cells (Figure 4B,C and Supporting Information S1: Figure S5A,B). Functional assays revealed that DTX3L depletion significantly inhibited cell proliferation (Figure 4D,E and Supporting Information S1: Figure S5C,D), impaired migration and invasion (Figure 4F and Supporting Information S1: Figure S5E), induced cell cycle arrest (Figure 4G and Supporting Information S1: Figure S5F), and increased apoptosis (Figure 4H and Supporting Information S1: Figure S5G). Furthermore, DTX3L knockdown led to a marked reduction in AKT phosphorylation levels (Figure 4I and Supporting Information S1: Figure S5H). In contrast, overexpression of DTX3L produced opposite effects, enhancing proliferation, migration, invasion, and AKT signaling (Supporting Information S1: Figure S6A–F). To evaluate the in vivo relevance of LINC00973 and DTX3L in NSCLC progression, xenograft tumor models were employed. Tumors derived from cells treated with siRNAs targeting either LINC00973 or DTX3L exhibited significant reductions in both volume and weight compared with control tumors (Figure 4J,K). IHC analysis further revealed decreased Ki‐67‐positive cell populations in tumors from the si‐LINC00973 and si‐DTX3L groups, indicating reduced proliferative activity (Figure 4L). Collectively, these results demonstrate that both LINC00973 and DTX3L play critical roles in promoting NSCLC tumor growth, at least in part through modulation of the AKT signaling pathway.

### 2.5 LINC00973 Inhibits the Ubiquitination and Degradation of DTX3L Protein

**FIGURE 5:**
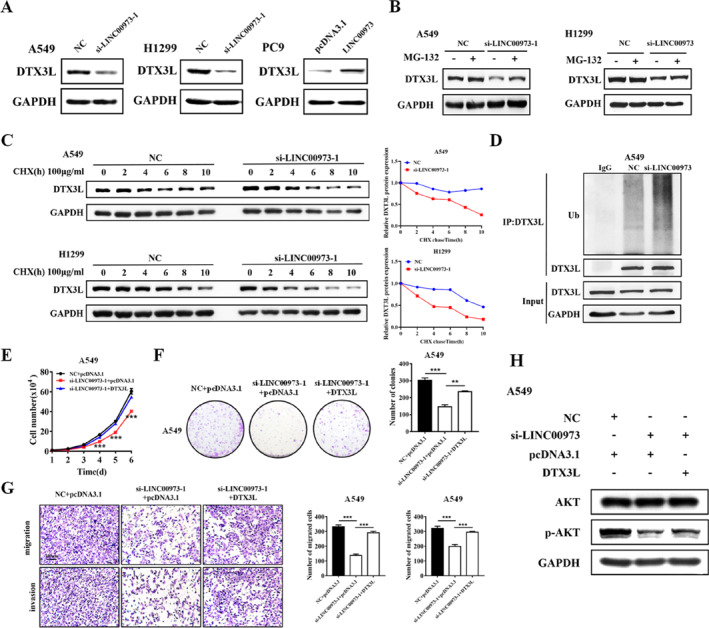
LINC00973 inhibits the ubiquitination and degradation of DTX3L protein. (A) Western blot analysis of DTX3L protein levels in NSCLC cells with LINC00973 knockdown or overexpression. (B) Western blot analysis of DTX3L protein levels in NSCLC cells with or without LINC00973 knockdown following treatment with the proteasome inhibitor MG‐132 (40 μM, 8 h). (C) Protein biosynthesis in NSCLC cells was blocked with 20 μg/mL of CHX. DTX3L protein levels in NSCLC cells with or without LINC00973 knockdown at different time points were examined by Western blot. (D) Ubiquitination assay showed DTX3L ubiquitination levels. NSCLC cells were co‐transfected with Ub and si‐LINC00973, treated with MG‐132, immunoprecipitated with an anti‐DTX3L antibody, and immunoblotted with an anti‐ubiquitin antibody. (E–H) Functional rescue experiments in NSCLC cells with LINC00973 knockdown and DTX3L overexpression, including CCK‐8 proliferation assays (E), colony formation assays (F), Transwell migration and Matrigel invasion assays (G), and Western blot analysis of related proteins (H). Data are shown as means ± SD (*p < 0.05; **p < 0.01; ***p < 0.001).

Western blot analysis revealed that LINC00973 knockdown decreased DTX3L protein levels, whereas LINC00973 overexpression produced the opposite effect, without significantly altering DTX3L mRNA expression (Figure 5A and Supporting Information S1: Figure S7A,B). Given that LINC00973 interaction increased DTX3L protein abundance without altering its transcription, we hypothesized that LINC00973 might enhance DTX3L stability by inhibiting its ubiquitin‐proteasome pathway‐mediated degradation. The results showed that MG‐132 effectively rescued DTX3L protein levels in LINC00973‐depleted NSCLC cells, indicating that LINC00973 stabilizes DTX3L by preventing its proteasomal degradation (Figure 5B). To further investigate the mechanism underlying DTX3L stabilization, cycloheximide (CHX) chase assays were performed. LINC00973 knockdown significantly reduced the half‐life of DTX3L protein compared with control cells, confirming that LINC00973 inhibits DTX3L degradation (Figure 5C). Analysis using PhosphoSitePlus software identified multiple potential ubiquitination sites on DTX3L (Supporting Information S1: Figure S7C), suggesting that LINC00973 may regulate DTX3L ubiquitination. Consistent with this, ubiquitination assays revealed that LINC00973 depletion markedly increased the levels of ubiquitinated DTX3L protein in NSCLC cells co‐transfected with ubiquitin and treated with MG‐132 (Figure 5D and Supporting Information S1: Figure S7D). To determine whether DTX3L mediates the oncogenic effects of LINC00973, rescue experiments were conducted. Overexpression of DTX3L in LINC00973‐depleted NSCLC cells effectively restored cell proliferation, migration, and invasion capacities (Figure 5E–G and Supporting Information S1: Figure S8A–C). Furthermore, DTX3L overexpression rescued the expression of key components of the AKT signaling pathway that had been downregulated by LINC00973 knockdown (Figure 5H and Supporting Information S1: Figure S8D). Taken together, these findings indicate that LINC00973 promotes NSCLC progression by stabilizing DTX3L protein through inhibition of ubiquitination‐dependent degradation, thereby sustaining AKT pathway activation.

### 2.6 Engineered Exosomes for the Delivery of LINC00973 siRNAs Suppress NSCLC Progression

**FIGURE 6:**
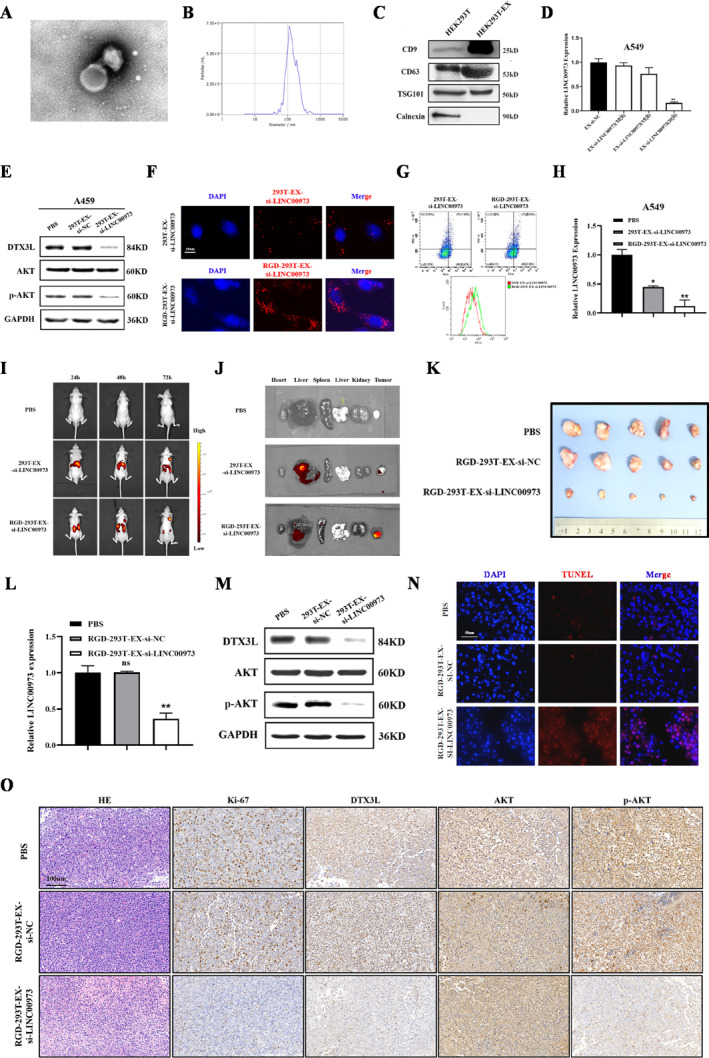
Engineered exosomes for targeted delivery of LINC00973 siRNA suppress NSCLC progression. (A and B) When examining exosomes derived from HEK293T cells, TEM (A) and NTA (B) were employed for comprehensive characterization. (C) Western blot analysis of exosomal marker proteins. (D) qRT‐PCR analysis of LINC00973 expression in NSCLC cells treated with exosome‐delivered siRNA. (E) Western blot analysis of DTX3L expression and AKT pathway activation in exosome‐treated NSCLC cells. (F and G) Uptake of DiI‐labeled exosomes by NSCLC cells examined by confocal microscopy (F) and flow cytometry (G). (H) qRT‐PCR analysis of LINC00973 expression in A549 cells treated with RGD‐293T‐EX‐si‐LINC00973. (I) The in vivo distribution of engineered exosomes in mice. (J) In vivo imaging of organs and tumors in mice 24–72 h after tail vein injection of engineered exosomes. (K) Observations on tumor images across various experimental groups. (L) qRT‐PCR analysis of LINC00973 expression in tumor tissues from different experimental groups. (M) Western blot analysis of DTX3L and *p*‐AKT protein levels in tumor tissues. (N) Representative images of TUNEL‐positive cells in tumor tissues from each experimental group. (O) IHC staining of indicated proteins in tumor tissues from different groups. Data are shown as means ± SD (**p < 0.01; ***p < 0.001; ns, not significant).

Since LINC00973 exerted a critical role in promoting NSCLC progression, an exosome‐based siRNA delivery approach was employed to target this lncRNA therapeutically. We characterized the exosomes isolated from HEK293T tool cells by transmission electron microscopy (TEM), nanoparticle tracking analysis (NTA), and Western blot analysis to detect exosomal markers (Figure 6A–C). The dose‐dependent suppression of LINC00973 by EX‐si‐LINC00973 in NSCLC cells was accompanied by reduced levels of DTX3L and *p*‐AKT (Figure 6D,E), consistent with earlier mechanistic findings. An RGD‐modified exosome platform, designated RGD‐293T‐EX‐si‐LINC00973, was subsequently constructed to enhance targeted delivery of LINC00973 siRNA. Both confocal microscopy and flow cytometry analyses confirmed that RGD modification significantly improved exosome internalization by NSCLC cells compared with unmodified exosomes (Figure 6F,G). Importantly, the RGD modification further enhanced these inhibitory effects, resulting in pronounced suppression of NSCLC cell proliferation, migration, and invasion compared with controls (Figure 6H and Supporting Information S1: Figure S9A–C). Subsequently, a subcutaneous tumor model in nude mice was utilized to evaluate the anti‐tumor effect of engineered exosomes, and the distribution of exosomes was tracked with IVIS. In vivo studies confirmed that the engineered exosomes accumulated at the tumor site and siRNA delivered by engineered exosomes significantly inhibited LLINC00973 expression and suppressed NSCLC growth (Figure 6I–L and Supporting Information S1: Figure S10A,B). Western blot analysis further demonstrated that the application of RGD‐293T‐EX‐si‐LINC00973 not only downregulated the protein level of DTX3L but also impaired the activation of the AKT signaling pathway (Figure 6M). IHC analysis further revealed decreased Ki‐67, DTX3L, and *p*‐AKT staining, while TUNEL assays indicated a substantial increase in apoptotic tumor cells (Figure 6N–O). Hematoxylin and eosin (H&E) staining and serological analyses confirmed minimal toxicity of the engineered exosomes to normal organs (Supporting Information S1: Figure S10C,D). Collectively, these results demonstrate that RGD‐modified exosomes can efficiently deliver siRNAs into NSCLC cells, suppress LINC00973 expression, and exert significant anti‐tumor effects in preclinical models.

### 2.7 In Vivo Anti‐LINC00973 siRNAs Within Self‐Assembled Exosomes Inhibit NSCLC Progression

To further enhance the therapeutic potential of targeting LINC00973 in NSCLC, a synthetic biology approach was employed to generate anti‐LINC00973 siRNAs in vivo within self‐assembled exosomes, as established in previous studies. The anti‐LINC00973 construct was designed to include a CMV promoter module, a pre‐miR‐155 backbone, and an anti‐LINC00973 siRNA‐encoding sequence. The pre‐miR‐155 framework is a well‐characterized and widely utilized carrier for siRNA synthesis [26]. In this system, the anti‐LINC00973 siRNA sequence was inserted into the guide strand position of the pre‐miR‐155 scaffold. To maximize the production of functional guide strands while minimizing undesired passenger strands, a CMV‐driven pre‐miRNA expression system was employed. This approach not only enhanced the yield of active siRNA guide strands but also reduced potential off‐target effects, thereby improving the specificity and efficacy of the therapeutic construct.

**FIGURE 7:**
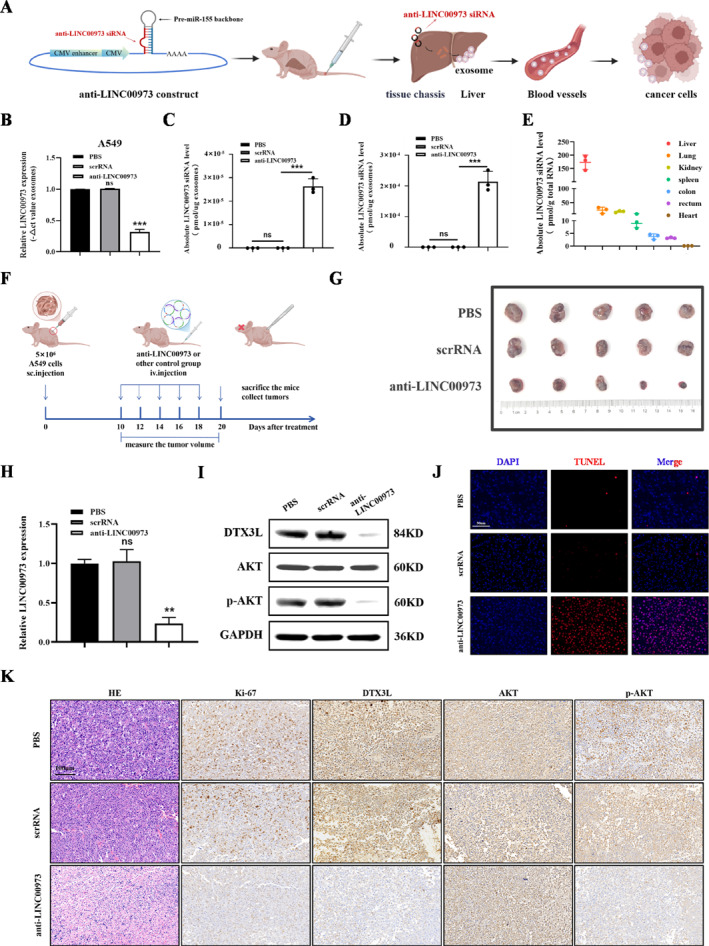
In vivo anti‐LINC00973 siRNAs within self‐assembled exosomes suppress NSCLC progression. (A) Schematic illustration of the in vivo assembly and delivery of anti‐LINC00973 siRNA‐loaded exosomes. (B) The relative expression level of LINC00973 after co‐incubation of exosomes with A549 cells. (C) Absolute expression level of anti‐LINC00973 siRNA in exosomes. (D) Absolute quantitative analysis of anti‐LINC00973 siRNA levels in serum exosomes from mice. (E) Following a single intravenous dose (5 mg/kg) of anti‐LINC00973 construct, the distribution kinetics of its siRNA was analyzed across major mouse organs. (F) Schematic overview of the exosome‐based antitumor therapeutic strategy. (G) Images of tumors from various experimental groups of mouse models, as indicated. (H) qRT‐PCR analysis of LINC00973 expression levels in tumor tissues from different experimental groups. (I) Protein levels of DTX3L and *p*‐AKT in tumor tissues were assessed with Western blot analysis. (J) Representative images of TUNEL staining of tumors in each group. (K) IHC staining showing the expression of indicated proteins in tumor tissues across different experimental groups. Data are shown as means ± SD (**p < 0.01; ***p < 0.001).

Upon uptake of the anti‐lncRNA construct by the liver, the CMV promoter facilitated the generation of anti‐lncRNA siRNA and drove the release of the siRNA payload into secretory exosomes. Previous studies by Fu et al. have demonstrated the feasibility and efficacy of this synthetic construct strategy [26]. Building on the critical role of LINC00973 in NSCLC progression, a synthetic construct was specifically engineered to target this lncRNA (Figure 7A), with its structural design illustrated in Supporting Information S1: Figure S11A. A corresponding negative control construct (scrRNA) was also prepared. Firstly, the sensitivity of absolute quantitative RT‐PCR assay was determined, with a cutoff cycle threshold (Ct) value of 35.03 for detecting anti‐LINC00973 siRNA (Supporting Information S1: Figure S11B). After transfecting anti‐LINC00973 siRNA into 293T cells, exosomes were collected by ultracentrifugation. Co‐incubation of these exosomes with A549 cells led to a significant reduction in LINC00973 expression (Figure 7B). Furthermore, absolute quantification via a standard curve confirmed a substantial increase in anti‐LINC00973 siRNA within the isolated exosomes (Figure 7C). Consistent results were observed in vivo, where tail vein injection of anti‐LINC00973 construct likewise led to elevated levels in subsequently collected serum exosomes (Figure 7D). Earlier reports indicated that self‐assembled plasmids primarily accumulate in the liver, lungs, colon, rectum, and kidney of mice at 6 h post‐tail vein injection, and our construct exhibited a similar distribution [23, 26]. In our study, the most prominent accumulation was also seen in the liver, and consistent with the prior findings, the signal was undetectable in heart tissue (Figure 7E).

After four rounds of treatment, tumors in mice receiving the anti‐LINC00973 construct exhibited significant reductions in both volume and weight compared with the scrRNA group (Figure 7F,G and Supporting Information S1: Figure S11C–E). Correspondingly, mRNA expression of LINC00973 and protein levels of DTX3L and *p*‐AKT were markedly decreased in tumors from the anti‐LINC00973 group relative to controls (Figure 7H,I). IHC and TUNEL staining further confirmed that anti‐LINC00973 treatment reduced tumor cell proliferation and increased apoptosis. Additionally, the number of tumor cells positive for DTX3L and *p*‐AKT was substantially lower in the anti‐LINC00973 group (Figure 7J,K). Furthermore, the anti‐LINC00973 siRNA group showed no obvious variations in the morphology and function of major organs (Supporting Information S1: Figure S11F,G). Collectively, these findings indicate that the anti‐LINC00973 construct can stimulate the liver to produce exosomes carrying functional siRNA, enabling effective systemic delivery and suppression of NSCLC tumor growth in vivo.

## 3 Discussion

LncRNAs are increasingly recognized as critical regulators in the development of various diseases, including cancer [27, 28, 29]. In this study, we identified LINC00973 as a potent facilitator of NSCLC progression. LINC00973 was significantly overexpressed in NSCLC tumor tissues, and elevated expression correlated with poor clinical outcomes, suggesting its potential as a prognostic biomarker. Interestingly, unlike many conventional biomarkers, LINC00973 expression did not correlate with TNM stage, indicating that it reflects intrinsic tumor aggressiveness. This feature may allow LINC00973 to stratify patients within the same pathological stage, particularly in early‐stage NSCLC, where accurately identifying individuals at high risk of recurrence remains a significant clinical challenge. Mechanistically, LINC00973 directly interacts with the E3 ubiquitin ligase DTX3L, inhibiting its ubiquitination and proteasomal degradation. This stabilization of DTX3L activates the AKT signaling pathway, thereby promoting NSCLC cell proliferation and metastasis both in vitro and in vivo. Based on the critical roles of LINC00973 in NSCLC development, we developed two strategies for targeted inhibition of LINC00973 through in vitro loading of siRNAs into engineered exosomes and in vivo generation of self‐assembled siRNA‐loaded exosomes in the liver. To enhance tumor targeting, the exosomes were engineered with RGD peptides, thereby improving their delivery to tumor sites and therapeutic efficacy. This target‐and‐deliver strategy ensured that the therapeutic siRNA was preferentially localized to the tumor, enhancing the target effects while reducing potential off‐target toxicity. Both approaches effectively suppressed LINC00973 in NSCLC cells and demonstrated significant anti‐tumor activity in mouse models. Collectively, these findings highlight LINC00973 as a critical driver of NSCLC progression and a promising therapeutic target, offering new avenues for prognostic assessment and targeted intervention in this disease.

Previous studies have demonstrated that the lncRNA LINC00973 exhibits elevated expression levels in multiple human malignancies, including colorectal cancer and clear‐cell renal cell carcinoma. A bioinformatics analysis by Guo and colleagues further demonstrated that LINC00973 expression is elevated in NSCLC tissues [30]. Despite these observations, the biological functions and underlying mechanisms of LINC00973 in cancer remain poorly understood. For instance, Wang et al. reported that LINC00973 promotes tumor growth by directly interacting with LDHA, thereby enhancing the Warburg effect in cancer cells [31]. Additionally, Shao et al. reported that the EGFR/Wnt signaling pathway upregulates LINC00973 by via β‐catenin activation, and that LINC00973 can function as a molecular decoy for miR‐216b (targeting CD55) and miR‐150 (targeting CD59). This mechanism ultimately contributes to tumor immune evasion through a complement inhibition‐dependent pathway [32]. Recent studies suggest that lncRNA can bind to specific proteins, thus regulating their structures and functions [33]. Consistent with the notion that lncRNAs can interact with specific proteins to modulate their structure and function, our study demonstrates that LINC00973 contributes to NSCLC progression by binding to and regulating DTX3L. This finding uncovers a novel mechanistic pathway underlying the oncogenic properties of LINC00973 in cancer. Further investigations into the complex regulatory networks involving LINC00973 are expected to provide new insights into its biological functions and potential as a therapeutic target.

DTX3L, also known as B‐lymphoma and BAL‐associated protein, is a Deltex family E3 ligase [34, 35]. Previous studies have highlighted the critical role of DTX3L in promoting tumorigenesis and cancer progression across multiple malignancies. For instance, in esophageal squamous cell carcinoma (ESCC), DTX3L has been shown to enhance cell proliferation and migration, as well as facilitate infiltration of M2 tumor‐associated macrophages (TAM) [36]. Beyond ESCC, DTX3L also contributes to prostate cancer (PCa) progression through its interaction with ARTD9, promoting cancer cell survival and proliferation; importantly, targeting DTX3L in PCa has been shown to improve the efficacy of chemotherapy and radiotherapy [37]. Furthermore, circular RNA circEYA3 alleviated radiation‐induced DNA damage and decreased radiosensitivity in hepatocellular carcinoma via the IGF2BP2/DTX3L axis [38]. Despite these insights, the role of DTX3L in NSCLC has remained largely unexplored. In the present study, we demonstrated that LINC00973 specifically interacts with DTX3L, stabilizing its protein by inhibiting ubiquitination and proteasomal degradation. This interaction suggests that LINC00973 may facilitate NSCLC progression, at least in part, through the post‐translational regulation of DTX3L. Notably, while LINC00973 expression remained comparable across stages, DTX3L and *p*‐AKT protein levels were strikingly elevated in advanced‐stage (III/IV) tumors. This stage‐dependent upregulation suggests enhanced pathway activity with disease progression. Furthermore, the strong positive correlation between DTX3L and *p*‐AKT levels within the same tissue specimens provides direct evidence for an active, clinically relevant molecular axis in human NSCLC.

In recent years, exosomes have emerged as versatile drug delivery systems in cancer therapy due to their excellent biocompatibility and unique physicochemical properties, enabling efficient transport of siRNAs, miRNAs, nanoantibodies, and small‐molecule drugs to target sites. For example, Zhang et al. encapsulated doxorubicin into exosome‐like nanovesicles from neutrophils and further conjugated them with superparamagnetic iron oxide nanoparticles. These engineered nanocarriers selectively accumulated in the tumor microenvironment, effectively inhibiting tumor progression and significantly prolonging survival in experimental mice [16]. Similarly, Chen et al. developed a dual‐targeting nanodelivery system using milk exosomes to target both tumor cells and macrophages simultaneously, successfully reprogramming TAMs and achieving substantial anti‐tumor effects [17]. Furthermore, RGD‐containing exosomes derived from mesenchymal stem cells have been shown to promote blood‐spinal cord barrier repair and enhance neurological recovery in mouse models of spinal cord injury [39]. Inspired by this, we developed an RGD‐modified engineered exosome system to deliver LINC00973‐targeting siRNA. This platform efficiently suppressed LINC00973 and DTX3L expression while concurrently inhibiting AKT pathway activation in tumor tissues. Consequently, it significantly impeded NSCLC tumor progression in vivo, highlighting the potential of RGD‐engineered exosomes carrying LINC00973 siRNA as a novel therapeutic strategy for NSCLC.

Given that ncRNAs account for over 90% of the human genome, RNAi therapy targeting ncRNAs holds significant importance for advancing disease treatment [40]. Building on previous studies that utilize in vivo self‐assembly siRNA strategies for the treatment of other diseases, we developed a biological construct using synthetic biology for in vivo self‐assembly and targeted delivery of siRNA for inhibition of LINC00973 [41]. This strategy reprogrammed the mouse liver as a tissue platform to harness the spontaneous synthesis capacity of the liver to encapsulate siRNA into exosomes. It showed potent anti‐tumor effects while exhibiting few toxic and side effects in mouse tumor models. Notably, this RNAi‐based therapeutic approach overcomes several limitations of conventional siRNA treatments, including toxicity, immunogenicity, and the complex manufacturing and purification challenges associated with exosome‐based delivery, offering a novel and effective method for NSCLC management.

Although our study reveals a previously unrecognized mechanism whereby LINC00973 interacts with and stabilizes DTX3L by inhibiting its ubiquitin‐mediated degradation, the precise molecular details of this interaction remain to be fully elucidated. Future investigations should aim to identify the specific regions within LINC00973 and DTX3L that mediate their binding, as well as the exact ubiquitination sites on DTX3L that are protected by LINC00973. Elucidating these structural and mechanistic details will provide deeper insights into this regulatory axis. Nevertheless, our findings unequivocally highlight the functional significance of the LINC00973/DTX3L/AKT pathway in promoting NSCLC progression.

**FIGURE 8:**
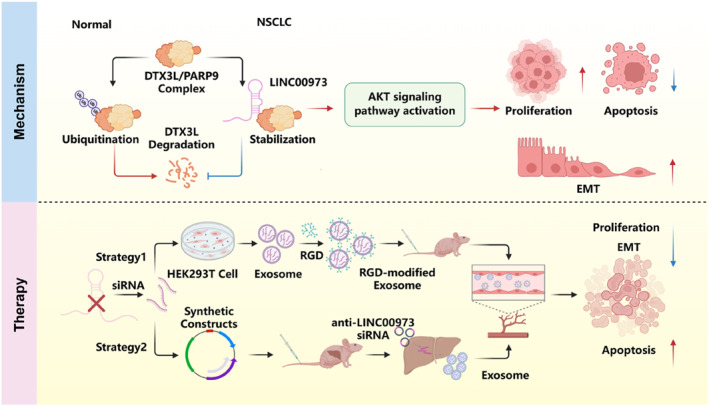
Proposed model for the oncogenic roles of LINC00973 in NSCLC progression and its potential in NSCLC therapy.

Collectively, we reported a new lncRNA, LINC00973, as a potent tumor‐promoting factor in NSCLC (Figure 8). Mechanistically, LINC00973 drives NSCLC progression by binding to DTX3L, preventing its ubiquitination and proteasomal degradation, and consequently activating the AKT signaling pathway. These findings expand our understanding of the molecular roles of lncRNAs in NSCLC and highlight LINC00973 as a promising candidate for both prognostic biomarker development and targeted therapeutic interventions in this malignancy.

LINC00973 promotes NSCLC progression by interacting with DTX3L, leading to activation of the AKT signaling pathway. Therapeutically, LINC00973 can be targeted by siRNA delivery strategies, including engineered exosomes for in vitro inhibition and in vivo self‐assembly of siRNAs within endogenous liver‐derived exosomes, thereby offering a potential treatment approach for NSCLC.

## 4 Materials and Methods

### 4.1 Clinical Samples

NSCLC tissues and matched normal tissue samples (*n* = 61) were collected from NSCLC patients who underwent surgical resection at Nantong Tumor Hospital between November 2015 and October 2016. The diagnosis of NSCLC was confirmed through imaging studies and histopathological evaluation. The study was approved by the Ethics Committee of Nantong Cancer Hospital, and written informed consent was obtained from all participants prior to sample collection.

### 4.2 Cell Culture

Human NSCLC cell lines A549, obtained from Co‐bioer Biosciences (Nanjing, China) and NCI‐H1299, sourced from the Cell Resource Center, IBMS, CAMS/PUMC (Beijing, China). Human NSCLC cell line PC9 and human HBE cell line were purchased from the Chinese Academy of Sciences Cell Bank (CASCB, Shanghai, China). NCI‐H1299, PC9, and HBE cells were cultured in RPMI‐1640 medium (Gibco, USA), whereas A549 cells were maintained in DMEM/F‐12 medium (BioInd, Israel). All culture media were supplemented with 10% fetal bovine serum (Excell, China) and 1% penicillin/streptomycin. Cells were incubated under standard conditions at 37°C in a humidified atmosphere containing 5% CO_2_.

### 4.3 RNA Sequencing

To investigate the potential roles of lncRNAs in the progression of NSCLC, genome‐wide RNA sequencing was performed on three pairs of NSCLC tumor tissues and their matched adjacent normal tissues. Tissue quality control, sample preparation, and lncRNA sequencing were carried out by OE Biotech (Shanghai, China).

### 4.4 RNA Extraction and Quantitative Real‐Time Polymerase Chain Reaction (qRT‐PCR)

Total RNA was extracted from cells or tissues using the TRIzol reagent following the manufacturer's instructions (Vazyme Biotech, Nanjing, China). Complementary DNA (cDNA) was then synthesized from the isolated RNA using a cDNA synthesis kit (Vazyme Biotech). The primer sequences used in this study are listed in Supporting Information S1: Table S2. qRT‐PCR was performed using SYBR Green PCR kits (Vazyme Biotech) on a QuantStudio 3 detection system (Thermo Fisher, USA). Relative gene expression levels were calculated using the 2^−ΔΔCt^ method, with U6 serving as the internal reference for normalization.

For specific small RNA detection, mature anti‐LINC00973 siRNA was reverse‐transcribed using a stem‐loop method (1st Strand cDNA Synthesis Kit, Vazyme) after gDNA removal. qPCR was performed with Universal SYBR Master Mix, forward primer and reverse primer. Absolute quantification was achieved using a synthetic RNA standard curve.

To establish a method for absolute quantification, a standard curve was generated using serially diluted synthetic single‐stranded LINC0097 siRNA, which was amplified by SYBR Green qRT‐PCR. The assay demonstrated consistent and efficient amplification across a wide dynamic range, with CT values from 13.87 to 35.03. The absolute concentration of anti‐LINC00973 siRNA in tissue samples was calculated by interpolating sample CT values against this standard curve. Results are expressed as the amount of anti‐LINC00973 siRNA per gram of total RNA isolated from each mouse tissue (pmol/g total RNA).

### 4.5 Western Blot

Protein samples were extracted from tissues or cells using RIPA lysis buffer (Thermo Fisher, USA) supplemented with protease inhibitors to prevent protein degradation. The lysates were separated on 10% SDS‐PAGE gels and subsequently transferred onto polyvinylidene fluoride membranes (Roche, Switzerland). Membranes were blocked in Tris‐buffered saline with 0.1% Tween‐20 (TBST) containing 5% bovine serum albumin for 1 h at room temperature, followed by overnight incubation with primary antibodies at 4°C. After washing, membranes were incubated with appropriate secondary antibodies for 2 h at room temperature. Following three washes with TBST (5 min each), protein bands were visualized using an ECL Western Blotting Kit (Vazyme Biotech).

### 4.6 Exosome Extraction and Identification

After 48 h of culture, conditioned medium was collected from HEK293T cells transfected with either si‐LINC00973 or si‐NC. Cells were maintained in DMEM supplemented with 10% exosome‐depleted fetal bovine serum. When cell confluence reached approximately 80%, the culture medium was replaced with fresh medium, and after 24 h, the supernatant was harvested. The collected medium was sequentially centrifuged at 300 × g for 20 min to remove floating cells, 2000 × g for 20 min to eliminate larger cellular debris, and 10,000 × g for 30 min to remove smaller particles. The resulting supernatant was pre‐cleared using a 0.22 μm filter (Millipore, MA, USA) and further concentrated with a 100 kDa ultrafiltration centrifugal tube. Exosomes were then isolated by ultracentrifugation using a SW 70 Ti swinging‐bucket rotor (Beckman Coulter, Shanghai, China) at 100,000 × g for 70 min, repeated twice. The isolated exosomes were characterized by TEM, NTA, and Western blot to confirm their morphology, size distribution, and marker protein expression.

### 4.7 Construction of Synthetic Constructs Targeting LINC00973

To generate the anti‐LINC00973 expression cassette, the anti‐LINC00973 siRNA sequence was cloned into the pre‐miR‐155 precursor backbone, with the CMV promoter positioned upstream of the pre‐miR‐155 scaffold. The recombinant plasmid was then cultured in LB medium containing 50 μg/mL spectinomycin at 37°C for 14–16 h. Following bacterial growth, plasmid DNA was extracted and purified using the EndoFree Maxi Plasmid Kit (Tiangen, China).

### 4.8 Cell Transfection

For functional studies, the LINC00973 overexpression plasmid was constructed and validated by BersinBio Company (Guangzhou, China). siRNAs specifically targeting the junctional region of LINC00973 were designed and synthesized by GenePharma (Shanghai, China). NSCLC cells were seeded in 6‐well plates and cultured overnight before transfection with Lipofectamine 2000 (Invitrogen, Carlsbad, CA, USA) following the manufacturer's instructions. After 4–6 h, the transfection medium was replaced with fresh culture medium, and the cells were further incubated for 24–48 h. The sequences of siRNAs used in this study are listed in Supporting Information S1: Table S3.

### 4.9 Cell Migration and Invasion Assays

For the cell migration assay, 5 × 10^4^ cells in 200 μL of serum‐free medium were seeded into the upper chamber of Transwell inserts (Corning Life Sciences, Suzhou, China). For the invasion assay, 1 × 10^5^ cells in 200 μL of serum‐free medium were added to Matrigel‐coated upper chambers (BD Biosciences). In both assays, 600 μL of complete medium was placed in the lower chamber as a chemoattractant. Cells were incubated at 37°C in a humidified atmosphere containing 5% CO_2_ for 48 h. After incubation, cells on the lower surface of the membrane were fixed with 1 mL of 4% paraformaldehyde at room temperature for 30 min and subsequently stained with 1 mL of 0.1% crystal violet for 15 min. Cells remaining on the upper surface of the membrane were gently removed with a cotton swab. After air‐drying, migrated or invaded cells were imaged under a microscope in three randomly selected fields per well, and the number of cells was counted manually.

### 4.10 Tagged RNA Affinity Purification (TRAP) Assay

Control and LINC00973‐overexpressing plasmids, along with a GST‐MS2 overexpression vector, were generated by Biosense (Guangzhou, China). Both LINC00973‐overexpressing and control plasmids contained MS2 stem‐loop sequences (LINC00973‐MS2 and MS2, respectively). NSCLC cells were co‐transfected with the LINC00973‐MS2 or MS2 vectors together with the GST‐MS2 vector to assemble the GST‐MS2‐LINC00973 complex. The complex was then captured using glutathione magnetic beads, and proteins specifically interacting with LINC00973 were identified by mass spectrometry and subsequently validated by Western blot analysis.

### 4.11 RNA Immunoprecipitation (RIP) Assay

RIP was performed using the Magna RIP RNA‐Binding Protein Immunoprecipitation Kit (Millipore, Bedford, MA, USA) according to the manufacturer's instructions. Cells were lysed in RIP lysis buffer supplemented with protease inhibitors and RNase inhibitors. The lysates were then incubated with magnetic beads pre‐bound with anti‐IgG, anti‐DTX3L, or anti‐PARP9 antibodies at room temperature for 1 h, followed by immunoprecipitation at 4°C overnight. After the procedure, RNA was purified from the immunoprecipitates and analyzed by qRT‐PCR.

### 4.12 LC‐MS/MS

Protein samples digested with proteases were analyzed by LC‐MS/MS to generate raw mass spectrometry data files. The resulting raw data were analyzed using Byonic software and searched against the UniProt *Homo sapiens* database to identify the proteins present.

### 4.13 Preparation of RGD‐293T‐EX

Exosomes (293T‐EX‐si‐NC/LINC00973) were isolated by ultracentrifugation as previously described. For RGD modification, 50 μg of RGD peptide (MCE, HY‐P0278) was incubated with exosomes in PBS at 37°C for 30 min, resulting in the formation of RGD‐293T‐EX‐si‐NC/LINC00973.

### 4.14 Mouse Tumor Model and In Vivo Imaging

Four‐ to six‐week‐old male BALB/c nude mice were obtained from the Experimental Animal Center of Jiangsu University and maintained under pathogen‐free conditions. All experimental procedures were approved by the Animal Ethics Review Board of Jiangsu University. The mice were randomly assigned to three groups (*n* = 5 per group) and subcutaneously injected with 5 × 10^6^ A549 cells transfected with si‐NC, si‐LINC00973, or si‐DTX3L. Tumor growth was monitored every 3 days, and tumor volumes were calculated using the formula: volume = 0.5 × (width^2^ × length).

To evaluate the targeted delivery of LINC00973 siRNA via engineered exosomes, 5 × 10^6^ A549 cells were subcutaneously implanted into nude mice, and tumor dimensions were measured every 3 days. Once tumor volumes reached approximately 100 mm^3^, mice were administered different RGD‐293T‐EX preparations via tail vein injection at a dose of 5 mg/kg every 3 days for a total of four treatments. For the in vivo self‐assembled LINC00973 siRNA encapsulated in exosomes, control (scrRNA) or experimental (anti‐LINC00973) constructs were injected via the tail vein every two days, for a total of four administrations.

Excised subcutaneous tumor tissues were collected and fixed in 4% paraformaldehyde. Samples were subsequently processed for histological and IHC analyses. H&E staining was performed to evaluate general tissue morphology, while IHC was used to assess the expression of specific proteins.

A subcutaneous tumor model was generated in mice by inoculating A549 cells. Once tumors had formed and grown for 7–10 days, mice received an intravenous injection of DiR‐labeled engineered exosomes. Whole‐body fluorescence imaging was performed at 24, 48, and 72 h post‐injection with the IVIS system. Following the final imaging time point, major organs and tumors from mice were collected for fluorescence imaging.

### 4.15 Analysis of Major Organ Function and Biochemical Indicators

Two days after the final treatment, mice were euthanized, and major organs, including the heart, liver, spleen, lungs, and kidneys, were collected for H&E histopathological analysis to evaluate potential toxic effects (Wuhan Servicebio Technology, China). Peripheral blood samples were also collected and centrifuged at 3000 × g for 10 min to obtain serum. Comprehensive serological analysis was then performed to assess key biochemical parameters, including liver function indicators (AST, ALT), kidney function markers (BUN, CREA), and myocardial enzyme profiles (LDH, CK).

### 4.16 Statistical Analysis

Statistical analyses were performed using SPSS 16.0 (SPSS, Chicago, IL, USA) and GraphPad Prism 10.0 (GraphPad Software, La Jolla, CA, USA). Depending on the experimental design, data were analyzed using Student's *t*‐test or one‐way ANOVA, as appropriate. Survival data were evaluated using Kaplan–Meier survival curves, and differences between groups were assessed with the log‐rank test. A *p* value of < 0.05 was considered statistically significant.

## Author Contributions

**Yanke Chen:** writing – original draft, conceptualization, methodology, investigation. **Yu Qian:** writing – original draft, conceptualization, methodology, data curation. **Jiayuan Shi:** validation. **Yi Wang:** methodology. **Tingyu Fu:** investigation. **Shuting Meng:** formal analysis. **Maoye Wang:** visualization. **Min Fu:** data curation. **Jiahui Zhang:** project administration. **Xiaoxin Zhang:** investigation. **Runbi Ji:** resources, software. **Jianmei Gu:** investigation, resources. **Xu Zhang:** conceptualization, methodology, funding acquisition, writing – review and editing, supervision. **Zhe‐Sheng Chen:** methodology, writing – review and editing, supervision. **Xiuqin Ma:** conceptualization, methodology, resources, project administration. **Xinjian Fang:** conceptualization, resources, methodology, supervision. All of the authors are aware of and agree to the content of the paper and are listed as co‐authors of the paper.

## Ethics Statement

Approval of the research protocol by an Institutional Review Board: N/A. Informed Consent: N/A. Registry and the Registration No. of the study/trial: N/A. Animal Studies: The Animal Use and Care Committee of Jiangsu University granted approval for all experiments involving animals (AP‐2023022703).

## Consent

Informed consent was obtained from all authors for the publication of this manuscript.

## Conflicts of Interest

The authors declare no conflicts of interest.

## Supporting information

Supporting Information S1

## Data Availability

No datasets were generated or analyzed during the current study.
