# Supplementary material for: LINC00973/DTX3L Axis Promotes Non‐Small Cell Lung Cancer Progression and Serves as a Therapeutic Target
Source: Smart Med. 2026 Feb 9;5(1):e70029. doi: 10.1002/smmd.70029 (PMC12893228; doi:10.1002/smmd.70029)
Supplement: Supplementary file 1 — Supporting Information S1 [file SMMD-5-e70029-s001.docx]

**Supplementary Materials**

**Table S1 LC-MS/MS analysis of potential proteins binding to LINC00973**

| **Protein** | **MS2** | **LINC00973-MS2** |
| --- | --- | --- |
| DTX3L | N/A | 64845000 |
| PARP9 | N/A | 18280333 |
| NAMPT | 8801666 | 53765000 |
| EDF1 | N/A | 33287666 |
| UBIP1 | N/A | 16231000 |
| TGM2 | N/A | 22320333 |
| E9PFF5 | N/A | 21314666 |

**Table S2 Primer sequences of target genes**

| **Gene name** | **Primer sequence** |
| --- | --- |
| U6 | F:5’-CTCGCTTCGGCAGCACA-3’  R:5’-AACGCTTCACGAATTTGCGT-3’ |
| GAPDH | F:5’-TGTGGGCATCAATGGATTTGG-3’  R:5’-ACACCATGTATTCCGGGTCAAT-3’ |
| β-ACTIN | F:5’-CACGAAACTACCTTCAACTCC-3’  R:5’-CATACTCCTGCTTGCTGATC-3’ |
| DTX3L | F:5’-AGCACCAAATACTTGTTGACGA-3’  R:5’-CACCAGACGGTGTTTCTGCTT-3’ |
| LINC00973 | F:5’-TTGAAGGCTTCCTGGTCTGAG-3’  R:5’-AGGCTTACATTCCAGCTGTGT-3’ |
| VCAM1 | F:5’-GGGAAGATGGTCGTGATCCTT-3’  R:5’-TCTGGGGTGGTCTCGATTTTA-3’ |
| MMP9 | F:5’-TGTACCGCTATGGTTACACTCG-3’  R:5’-GGCAGGGACAGTTGCTTCT-3’ |
| ITGA7 | F:5’-CTGACTCCATGTTCGGGATCA-3’  R:5’-CACCTGTGAAGGTTTGGCG-3’ |
| IGF2 | F:5’-GTGGCATCGTTGAGGAGTG-3’  R:5’-CACGTCCCTCTCGGACTTG-3’ |
| SCD | F:5’-TCTAGCTCCTATACCACCACCA-3’  R:5’-TCGTCTCCAACTTATCTCCTCC-3’ |
| YBX2 | F:5’-GCTGGCAATCCAAGTCCTG-3’  R:5’-TCAAATTCCACAGTCTCCCCAT-3’ |
| MMP19 | F:5’-TGTACCGCTATGGTTACACTCG-3’  R:5’-GGCAGGGACAGTTGCTTCT-3’ |
| KLK10 | F:5’-CAAGGCGAACGGATGAGCA-3’  R:5’-GAGCACAGCGGTAGGGAAG-3’ |
| ITGA2 | F:5’-GGGAATCAGTATTACACAACGGG-3’  R:5’-CCACAACATCTATGAGGGAAGGG-3’ |
| CDH4 | F:5’-TCCGGTCCGACAAAGACAATG-3’  R:5’-CATGGGCCTTGTGACGTACAT-3’ |
| ABCG4  anti-LINC00973 siRNA | F:5’-CCGTGGACATCGAGTTCGTG-3’  R:5’-TGAGAGGCACTTGAGAAGGGT-3’  RT:5’-GTCGTATCCAGTGCAGGGTCCGAGGTATTCGCACTGGATACGACAAATGA-3’  F:5’-CGCGGCACGACTTCTGG-3’  R:5’-AGTGCAGGGTCCGAGGTATT-3’ |

**Table S3 The sequences of siRNAs**

| **Gene name** | **Sense** | **Antisense** |
| --- | --- | --- |
| si-LINC00973-1 | GCUCCUGAGCUGACACUAATT | UUAGUGUCAGCUCAGGAGCTT |
| si-LINC00973-2 | GCACGACUUCUGGUCAUUUTT | AAAUGACCAGAAGUCGUGCTT |
| si-LINC00973-3 | CCUCAGUACUUUACUGAAUTT | AUUCAGUAAAGUACUGAGGTT |
| si-DTX3L-1 | GCAUUCUGUUUGAAUCCAATT | UUGGAUUCAAACAGAAUGCTT |
| si-DTX3L-2 | GCCUUUCAACAUGCCUCAUTT | AUGAGGCAUGUUGAAAGGCTT |
| Si-DTX3L-3 | CCAGACUUCCUAUGGUAUUTT | AAUACCAUAGGAAGUCUGGTT |

**Figure S1. LINC00973 expression in different stages of NSCLC patients.**

A. The top five upregulated lncRNAs in NSCLC tissues compared to normal tissues. B. QRT-PCR analyses of LINC00973 expression in paired tumor and normal tissues from 61 NSCLC patients. C. QRT-PCR analyses of LINC00973 expression in different stages of NSCLC patients. D. LINC00973 expression in different stages of NSCLC patients analyzed with TCGA data. Data are shown as means ± SD (****P*<0.001; ns, not significant)

**Figure S2. Knockdown of LINC00973 suppresses NSCLC growth and metastasis *in vitro.***

A. The efficiency of LINC00973 knockdown in H1299 cells. B-F. Cell growth curves (B), colony formation assays (C), Transwell migration and Matrigel invasion assays (D), flow cytometric analyses of cell cycle (E) and cell apoptosis (F) for LINC00973 knockdown H1299 cells. G. Western blot analyses of the expression of c-Myc, Cyclin D1 and EMT markers in H1299 cells. Data are shown as means ± SD (**P*<0.05; ***P*<0.01; ****P*<0.001).

**Figure S3. Overexpression of LINC00973 promotes NSCLC cell growth and metastasis.**

A.The efficiency of LINC00973 overexpression in PC9 cells. B-E. Cell growth curves (B), colony formation assays (C), Transwell migration and matrigel invasion assays (D), and cell cycle distribution (E) for LINC00973 overexpressing PC9 cells. F. Western blot analyses of the expression of c-Myc, Cyclin D1, and EMT markers in PC9 cells. Data are shown as means ± SD (**P*<0.05; ****P*<0.001).

**Figure S4. Overexpression of LINC00973 promotes NSCLC cell growth and metastasis.**

A. TCGA and GTEx databases analysis of DTX3L expression in lung cancer tissues and paired normal ones. B. Correlation analyses of DTX3L expression and NSCLC patients’ overall survival in the GEPIA database. C. Comparison of DTX3L and p-AKT expression by IHC between early- (Stage I) and advanced-stage (Stage III/IV) NSCLC patients.

**Figure S5. Knockdown of DTX3L suppresses NSCLC growth and metastasis in *vitro*.**

A-B. The knockdown of DTX3L mRNA and protein expression was demonstrated by qRT-PCR (A) and Western blot (B). C-G. Cell growth curves (C), colony formation assays (D), Transwell migration and matrigel invasion assays (E), cell cycle distribution (F), and flow cytometric analyses of cell apoptosis (G) for LINC00973 knockdown H1299 cells. H. Western blot analyses of the expression of AKT pathway proteins in H1299 cells. Data are shown as means ± SD (**P*<0.05; ***P*<0.01; ****P*<0.001).

**Figure S6. DTX3L overexpression facilitates NSCLC progression.**

A-B. The efficiency of DTX3L overexpression in H1299 cells. C-F. Cell growth curves (C), colony formation assays (D), transwell migration and matrigel invasion assays (E), cell cycle distribution (F) for LINC00973 overexpressing H1299 cells. Data are shown as means ± SD (**P*<0.05; ***P*<0.01;****P*<0.001).

**Figure S7. LINC00973 inhibits DTX3L ubiquitination and degradation.**

A. QRT-PCR assay for the mRNA levels of DTX3L in LINC00973 knockdown or overexpression NSCLC cells. B. Western blot assays for the protein levels of DTX3L in LINC00973 knockdown or overexpression NSCLC cells. C. PhosphoSitePlus database was applied to predict post-translational modification sites of DTX3L protein. D. Western blot assay for the ubiquitination of DTX3L protein in NSCLC cells treated with control and si-LINC00973. Data are shown as means ± SD (ns, not significant).

**Figure S8. Overexpression of DTX3L reverses the roles of knockdown LINC00973.**

A-D. CCK8 (A), colony formation (B), transwell migration and matrigel invasion (C), and western blot (D) assays for NSCLC cells with LINC00973 knockdown and DTX3L overexpression. Data are shown as means ± SD (***P*<0.01; ****P*<0.001).

**Figure S9. Engineered exosomes for the delivery of siRNAs targeting LINC00973 suppresses NSCLC progression.**

A-C. CCK8 (A), colony formation (B), transwell migration and matrigel invasion (C) assays for RGD-293T-EX-si-LINC00973 treated NSCLC cells. Data are shown as means ± SD (****P*<0.001; ns, not significant).

**Figure S10. The functions of major organs in mice treated with engineered exosomes.**

A. Imaging analysis of organs and tumors in mice 24-72 hours after tail vein injection of engineered exosomes. B. The progression of tumors in various mouse model groups, including tumor growth curves, volumes, and final weights. C. Histological analyses were performed on the sections of major organs. D. The effects of treatment on the liver (ALT and AST), kidney (BUN and CREA), and heart (LDH and CK) functions in mice were evaluated by blood biochemical analyses. Data are shown as means ± SD (****P*<0.001; ns, not significant).

**Figure S11.** **The functions of major organs in mice treated with anti-LINC00973.**

A. Map of the anti-LINC00973 siRNA construct. B. The dynamic range of absolute RT-PCR detection was determined using a synthetic anti-LINC00973 siRNA standard, which was serially diluted over six orders of magnitude. The corresponding CT values were plotted against the input log10(copies) to generate a standard curve. C-E. The progression of tumors in various mouse model groups, including tumor growth curves (C), volumes (D), and final weights (E). Data are shown as means ± SD. F. HE staining of major organs in mice of different groups. G. Blood biochemical tests of major organs of mice in different groups. Data are shown as means ± SD (ns, not significant).
